# Detected troponin elevation is associated with high early mortality after lung resection for cancer

**DOI:** 10.1186/1749-8090-1-37

**Published:** 2006-10-23

**Authors:** Eric Lim, Li Li Choy, Lydia Flaks, Shafi Mussa, Fillip Van Tornout, Marc Van Leuven, G Wyn Parry

**Affiliations:** 1 Department of Thoracic Surgery, Norfolk and Norwich University Hospital, Norwich, UK; 2 Department of Thoracic Surgery, Papworth Hospital, Papworth Everard, Cambridge CB3 8RE, UK

## Abstract

**Background:**

Myocardial infarction can be difficult to diagnose after lung surgery. As recent diagnostic criteria emphasize serum cardiac markers (in particular serum troponin) we set out to evaluate its clinical utility and to establish the long term prognostic impact of detected abnormal postoperative troponin levels after lung resection.

**Methods:**

We studied a historic cohort of patients with primary lung cancer who underwent intended surgical resection. Patients were grouped according to known postoperative troponin status and survival calculated by Kaplan Meier method and compared using log rank. Parametric survival analysis was used to ascertain independent predictors of mortality.

**Results:**

From 2001 to 2004, a total of 207 patients underwent lung resection for primary lung cancer of which 14 (7%) were identified with elevated serum troponin levels within 30 days of surgery, with 9 (64%) having classical features of myocardial infarction.

The median time to follow up (interquartile range) was 22 (1 to 52) months, and the one and five year survival probabilities (95% CI) for patients without and with postoperative troponin elevation were 92% (85 to 96) versus 60% (31 to 80) and 61% (51 to 71) versus 18% (3 to 43) respectively (p < 0.001).

T stage and postoperative troponin elevation remained independent predictors of mortality in the final multivariable model. The acceleration factor for death of elevated serum troponin after adjusting for tumour stage was 9.19 (95% CI 3.75 to 22.54).

**Conclusion:**

Patients with detected serum troponin elevation are at high risk of early mortality with or without symptoms of myocardial infarction after lung resection.

## Background

Postoperative myocardial infarction is not a well studied topic after lung surgery. From the few reports in the literature, the frequency to range between 0.7% to 2% [1,2], but it is difficult to determine an exact standarized incidence, in part due to differences in postoperative presentation, differences in EKG interpretation and a variation in the choice of serum cardiac markers.

Recently, the diagnostic criteria for myocardial infarction has evolved from a history of chest pain, EKG changes and a rise in serum cardiac markers, to an emphasis on serum cardiac markers (in particular serum troponin) as the primary diagnostic modality [3]. The normal postoperative profile of cardiac markers has only recently been established. In contrast to CK-MB, both cardiac troponin T and I are unaffected by routine thoracic (non-cardiac) surgery [4], supporting troponin as the cardiac marker of choice in this setting.

To evaluate the clinical utility of troponin assay after lung surgery, we set out to assess the frequency of classical supporting features of myocardial infarction and to establish the long term prognostic impact of detected abnormal postoperative troponin levels in patients who underwent lung resection for primary lung cancer.

## Methods

We studied a historic cohort of patients from 2001 to 2004 (the start date reflects the availability of troponin assay at our institution) with known or suspected primary lung cancer who underwent intended surgical resection. As cervical mediastinoscopy and biopsies were performed prior to thoracotomy for all patients with clinically suspected N2 disease, additional lymph node sampling was only undertaken in selected patients with unexpected or suspicious findings during thoracotomy. Staging was performed according to Mountain 1997 [5]. We excluded patients that did not prove to have primary lung cancer on histopathologic examination.

### Data acquisition

Patients were identified from our hospital National Health Service (NHS) database of Office of Population, Censuses and Surveys – Classification of Surgical Operations and Procedures codes (OCPS), and data obtained from case notes and histopathology reports. Survival was documented as the date of last follow up in a hospital outpatient clinic, and mortality documented as the date of death from patient records or the NHS strategic tracing service (NHS, UK).

### Troponin assay

Troponin I was analysed using the Access^® ^AccuTnI™ troponin I assay (Beckman Coulter Inc., CA, USA) with 0.04 μg/l as the upper reference limit of normal. The pathology database was screened for each patient individually to determine if a troponin sample was requested. Troponin levels of 0.04 μg/l or more within 30 days of surgery were classified as elevated in this study. Patients were grouped according to the presence of *known *post operative troponin elevation, and patients with normal or no known postoperative troponin levels were classified as "no troponin elevation".

### Statistical analysis

Categorical data are presented as frequency (%) and continuous data as mean with standard deviation (SD) or median with interquartile range (IQR). Comparisons of baseline categorical data between the two groups were made using Fisher's exact test and continuous data compared using two tailed t-test.

Parametric and non-parametric survival analyses were applied. Actuarial survival was estimated using the Kaplan-Meier method and compared using the Log-rank test. to Parametric survival analysis (log-logistic accelerated failure time model) was used to model the hazard function, ascertain the individual contribution of factors associated with survival and to compare the risk adjusted survival between the two groups. The survival analysis was repeated with non-parsimonious propensity score multivariable adjustment, and one-to-one (nearest neighbor) matching on age, sex, tumour stage, nodal status and operation extent. Statistical analyses were performed using R version 2.0.0 [6].

## Results

From 1^st ^January 2001 to 1^st ^January 2004, a total of 231 patients underwent lung resection for the presumed diagnosis of primary lung cancer. We excluded 26 patients who did not prove to have primary lung cancer. Of the 205 patients remaining, 41 had a troponin sample requested, 19 were elevated, of which 14 (7%) were elevated within 30 days of surgery. The remaining 5 patients in which the troponin levels were detected as elevated more than 30 days from surgery were analysed in the no troponin elevation group. The mean age (SD) of the cohort was 66 (11) years, and 133 (65%) were men. The baseline characteristics of the two groups are summarised in table 1.

**Table 1:** Baseline characteristics by group

	No troponin elevation	Troponin elevation	P value
Number	191	14	
Mean age, yrs (SD)	66 (11)	72 (5)	0.001
Men, n(%)	123 (64)	10 (71)	0.774
			
*Operation extent*			
Pneumonectomy, n(%)	45 (24)	5 (36)	
Lobectomy, n(%)	135 (70)	8 (57)	0.424
Other, n(%)	12 (6)	1 (7)	
			
*Cell type*			
Squamous, n(%)	83 (43)	7 (50)	
Adenocarcinoma, n(%)	68 (36)	5 (36)	
Large cell, n(%)	6 (3)	0 (0)	0.902
Mixed, n(%)	12 (6)	1 (7)	
Other, n(%)	24 (12)	1 (7)	
			
*T category*			
T1, n(%)	42 (22)	2 (14)	
T2, n(%)	112 (58)	10 (72)	
T3, n(%)	14 (7)	1 (7)	0.955
T4, n(%)	6 (3)	0 (0)	
Tx, n(%)	19 (10)	1 (7)	
			
*N category*			
N0, n(%)	111 (58)	7 (50)	
N1, n(%)	46 (24)	5 (36)	
N2, n(%)	17 (8)	1 (7)	0.811
Nx, n(%)	19 (10)	1 (7)	

**Figure 1:**
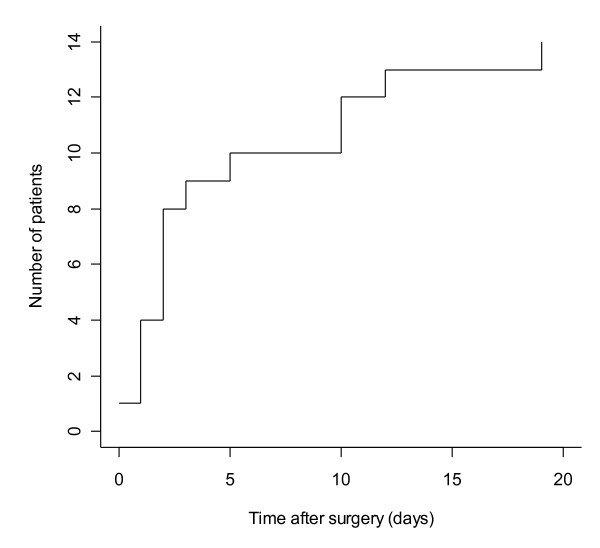
Cumulative frequency plot of time to detection of troponin elevation.

Of the 14 patients with elevated postoperative troponin levels, the mean peak level was 0.936 μg/l, and the majority (10/14) were detected within the first 5 postoperative days (Figure 1). In total, 5 had a preoperative history of stable ischaemic heart disease (4 had angina, 1 had a history of previous myocardial infarction). The main documented indication for postoperative troponin estimation was atrial fibrillation in 6 patients (department policy for screening patients with new onset postoperative atrial fibrillation), investigation of low blood pressure in 4 patients, postoperative chest pain in 2 patients and investigation of dyspnea in 2 patients. The revised definition for myocardial infarction (enzyme elevation accompanied by either supportive ECG changes or chest pain) was achieved in 9 patients (64%), with 5 fulfilling the criteria for ST elevation myocardial infarction and 3 fulfilling the criteria for non-ST-elevation myocardial infarction (one patient with chest pain and troponin elevation did not have any ECG changes of ischaemia).

**Figure 2:**
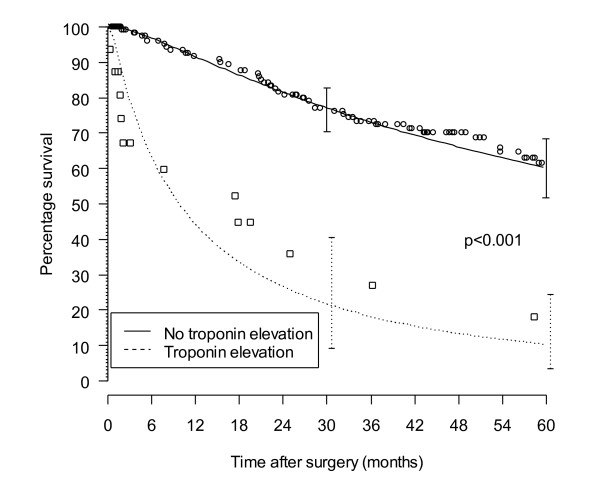
**Survival by postoperative troponin status**. Kaplan Meier estimates are represented by circles in the group with no known troponin elevation, and squares in the group with known troponin elevation. The lines are the fitted parametric survival estimates.

The median time to follow up (interquartile range) was 22 (1 to 52) months, with the one and five year survival probabilities (95% CI) for patients without and with postoperative troponin elevation as 92% (85 to 96) versus 60% (31 to 80) and 61% (51 to 71) versus 18% (3 to.43) respectively (p < 0.001, Figure 2).

The univariable prognostic predictors are summarised in table 2, and postoperative troponin elevation and T stage remained as independent predictors of mortality in the final multivariable model (table 3). The acceleration factor for mortality in patients with known postoperative troponin elevation was exp^-(-2.218)^, corresponding to 9.19 (95% CI 3.75 to 22.54).

**Table 2:** Univariable predictors of mortality

Variable	Coefficient (α)	SE	P value
Troponin elevation	-0.016	0.015	< 0.001
			
Age, per yr	-0.016	0.015	0.300
Male sex	-0.200	0.298	0.503
			
*T category*			
T1	1.000		
T2	-0.475	0.393	0.227
T3	-1.080	0.566	0.056
T4	-1.141	0.684	0.095
Tx	1.119	0.721	0.120
			
*N category*			
N0	1.000		
N1	-0.128	0.304	0.675
N2	-0.313	0.486	0.519
Nx	1.555	0.671	0.020
			
*Operation*			
Pneumonectomy	1.000		
Lobectomy	0.493	0.306	0.107
Other resection	0.948	0.563	0.093
			
*Cell type*			
Squamous	1.000		
Adenocarcinoma	-0.057	0.303	0.850
Large cell	-1.038	0.797	0.193
Mixed	-0.228	0.589	0.699
Other	1.109	0.584	0.058

*Exponentiation of the negative of the coefficient, exp^(-α) ^will give the acceleration factor

**Table 3:** Multivariable predictors of mortality

Variable	Coefficient (α)	SE	P value
Troponin elevation	-2.218	0.458	< 0.001
			
T0	1.000		
T2	-0.425	0.379	0.263
T3	-1.196	0.527	0.023
T4	-1.291	0.638	0.043
Tx	0.856	0.662	0.196

*Exponentiation of the negative of the coefficient, exp^(-α) ^will give the acceleration factor

**Figure 3:**
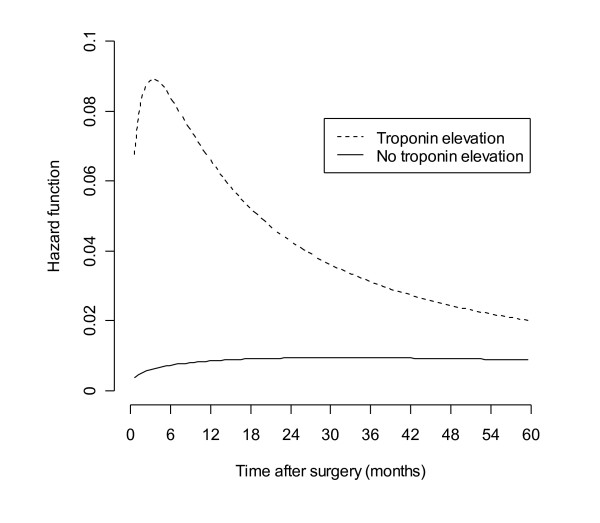
Hazard function by postoperative troponin status.

For patients with known troponin elevation, the instantaneous risk of death (hazard function, Figure 3) was highest in the first six months before gradually declining. This was in contrast to patients without (known) elevated postoperative levels where a small initial rise was followed by a relatively constant hazard for death.

When propensity score multivariable adjusted analysis as performed the results remained highly significant for early mortality in patients with known elevated troponin elevation (-2.30, p < 0.001) with a non-significant p-value for the coefficient for the propensity score (p = 0.11). The results remained robust even with one-to-one matching of the 14 patients with known elevated troponin levels to controls (-2.06, p < 0.001).

## Discussion

Normally, troponin is not detectable in the serum after lung surgery [4,7]. The results from our study reveal the prognostic impact of detected elevated postoperative serum troponin levels, which is associated high risk of early death.

The relationship to myocardial infarction is more difficult to delineate, as 36% (5/14) of patients with elevated serum troponin levels did not have chest pain or EKG evidence of ischemia. After thoracic surgery, we routinely use thoracic epidural analgesia supplemented with non-opiate oral analgesics. We believe that this practice is common and potentially attenuates symptoms of ischaemic chest pain. In addition, it might be more difficult for patients to distinguish between new onset chest pains against the background of thoracotomy wound pain. Classical ST-elevation on EKG was present in only 5 (36%) patients. Therefore, it can be difficult to establish a diagnosis of postoperative myocardial infarction in the setting of thoracic surgery.

Serum troponin levels may be useful in this regard, apart from greater cardio-specificity (compared to CK-MB), minute elevations were able to convey long term prognostic information. The upper reference limit (0.04 μg/l) in our study was five times lower than the reference limit of 0.20 μg/l required by the assay used in Vikenes et al. [4].

On one end of the spectrum of severity of myocardial infarction, myocardial necrosis detected on isolated serum troponin elevation may be a specific method to identify patients at a high risk of early death (within six months of surgery). Established postoperative myocardial infarction lies on the other extreme of the spectrum, and raises the 30-day mortality by an odds ratio of 32 [2]. The longer term consequences and impact on survival have not been quantified.

### Clinical implications

We highlight a potential use for serum troponin as a screening investigation to identify patients with a poor prognosis, regardless of further symptoms of myocardial infarction. This is consistent with observation that isolated troponin elevation is an independent predictor of mortality across the spectrum of the chest pain syndromes [8]. In total 6 (43%) patients were identified as part of a screening policy for new onset atrial fibrillation, suggesting that this may be a common presentation of postoperative myocardial infarction or (more accurately) myocardial necrosis without clinical evidence of infarction.

An unfortunate corollary of the retrospective nature of our study is the inability to confidently identify the patient risk factors that are associated with troponin elevation. Although patients with postoperative troponin elevation were significantly older, age alone is not a powerful discriminating feature. At a rate of 7% (95% CI 4 to 11) and estimated 9 fold acceleration in the risk of death, the clinical utility of serum troponin estimation after lung resection appears to be an important topic for further study. Importantly, only 5 of the 14 patients had a preoperative history of ischaemic heart disease, of which all had symptoms of stable angina and good performance status, not meeting the accepted criteria for further cardiovascular screening [9] The results of our study highlight the limitations of such recommendations, and suggest the need for further work in this area.

To date, there are no guidelines that address the management of myocardial infarction specifically after thoracic surgery. General guidelines for non-cardiac surgery stress the need for medical management (aspirin, beta-blockers, ACE inhibitors) and risk factor modification for secondary prevention [9]. Despite these efforts, survival in our cohort remained poor. Not all options are available, as thrombolysis is contraindicated after major thoracic surgery. Further work is required to determine if a more aggressive approach (primary angioplasty and stenting) could improve outcome, however, primary percutaneous coronary intervention also requires the use of anti-platelet agents, heparin and will be limited only to institutions where this facility is available.

### Potential limitations

The main limiting factors are the retrospective study design on a relatively small group rendering our study susceptible to bias. Unfortunately, sample size was limited by the relatively recent introduction of troponin assay at our institution. We have assumed that atrial fibrillation was associated with myocardial necrosis as we have found no evidence to suggest that atrial fibrillation alone elevates serum troponin independently of myocardial necrosis. However, our findings are not altered in respect of this relationship. Although we were not able to adjust for preoperative lung function in our analyses, the mean preoperative FEV_1 _of 2.2 l in patients in the troponin elevation arm was not different from our usual preoperative patient population.

### Future directions

It is clear that serum troponin is rapidly becoming the cardiac marker of choice, and prospective studies on unselected patients will be able to more accurately establish its utility as a prognostic marker. Further work would be able to determine if elevated troponin levels result from procedures that involve the pericardium (intra pericardial pneumonectomies), and ascertain if it carries similar prognostic implication.

## Conclusion

After lung surgery, approximately a third of patients with troponin elevation do not have classical symptoms of myocardial infarction. Atrial fibrillation was a common presenting feature leading to the identification of elevated serum troponin. Patients with detected serum troponin elevation are at high risk of early mortality after lung resection for primary lung cancer.
